# Evaluation of the Performance of an Artificial Intelligence-Based Classification Model for Pediatric Maxillofacial Morphology †

**DOI:** 10.3390/diagnostics15232958

**Published:** 2025-11-21

**Authors:** Hiroki Sato, Akane Ueda, Camila Tussie, Sophie Kim, Yukinori Kuwajima, Emiko Kikuchi, Shigemi Nagai, Kazuro Satoh

**Affiliations:** 1 Division of Orthodontics, Department of Developmental Oral Health Science, School of Dentistry, Iwate Medical University, 1-3-27 Chuo-dori, Morioka 020-8505, Japan; hiroki@iwate-med.ac.jp (H.S.); uakane@iwate-med.ac.jp (A.U.); ykuwaji@iwate-med.ac.jp (Y.K.); eaomatsu@iwate-med.ac.jp (E.K.); 2 Division of Orthodontics, Department of Developmental Biology, Harvard School of Dental Medicine, 188 Longwood Avenue, Boston, MA 02115, USA; camitussie@hsdm.harvard.edu; 3 Division of Orthodontics, Department of Regenerative and Reconstructive Sciences, UCLA School of Dentistry, 714 Tiverton, Los Angeles, CA 90095, USA; sophie@dentistry.ucla.edu; 4 Department of Restorative Dentistry and Biomaterials Sciences, Harvard School of Dental Medicine, 188 Longwood Avenue, Boston, MA 02115, USA; shigemi_nagai@hsdm.harvard.edu

**Keywords:** maxillofacial morphology, artificial intelligence, random forest, pediatrics

## Abstract

**Background/Objectives**: Accurate assessment of craniofacial morphology is essential for orthodontic diagnosis and treatment planning. The Sassoni classification provides a useful framework for categorizing craniofacial morphology into nine groups but lacks standardized clinical criteria. This study developed an AI model to classify pediatric craniofacial morphology based on the Sassoni classification using lateral cephalometric radiographs and evaluated its agreement with classifications made by orthodontists. **Methods**: Data from 300 pediatric patients aged 6 to 10 years were analyzed. Nine cephalometric measurements and patient gender were used as input features. Three orthodontists classified morphology based on the Sassoni classification. Random forest (RF), logistic regression (LR), and support vector classification (SVC) models were trained and evaluated using 10-fold cross-validation. **Results**: The Random Forest (RF) model demonstrated the highest accuracy (RF: 0.907 ± 0.051, LR: 0.837 ± 0.057, SVC: 0.770 ± 0.055). It also outperformed the other two models in terms of F1 score, sensitivity, and positive predictive value, showing the best overall classification performance. The most influential feature was the ANB angle, while gender had minimal impact. **Conclusions**: The RF-based AI model demonstrated high accuracy in pediatric maxillofacial classification. Performance may be further improved with larger datasets and more balanced case distributions.

## 1. Introduction

In recent years, artificial intelligence (AI) has increasingly been applied in healthcare to improve diagnostic accuracy [1,2].

Within orthodontics, research has focused on developing AI models for diagnostic and treatment planning purposes. One study used machine learning (ML) models trained on lateral cephalometric radiographs (lateral cephalograms) to determine the need for orthognathic surgery or tooth extraction [3].

In recent years, various applications of AI have been investigated, such as automatic landmark identification on cephalometric radiographs [4], prediction of three-dimensional facial morphology following orthognathic or orthodontic treatment [5], and other emerging approaches in orthodontic research [6,7].

Accurate diagnosis, the establishment of clear treatment goals, and effective treatment planning are all essential in orthodontics. For children in the growth and development phase, clinicians must account for jaw growth direction and treatment response in relation to the type of malocclusion. Accordingly, precise evaluation of maxillofacial morphology is critical when formulating a treatment plan. Lateral cephalometric analysis is widely used for assessing malocclusion and maxillofacial morphology and is considered a fundamental diagnostic tool in orthodontics. Various methods exist for categorizing maxillofacial morphology, most of which are based on lateral cephalometric analysis items.

The Sassouni classification defines maxillofacial morphology using horizontal and vertical relationships. Horizontally, skeletal Class I represents a balance between the upper and lower jaws, skeletal Class II reflects maxillary protrusion or mandibular retrusion, and skeletal Class III represents mandibular protrusion or maxillary retrusion. Vertically, patients are classified as short frame, medium frame, or long frame based on facial height. Combining these horizontal and vertical categories yields nine classification patterns [8].

Ricketts’ analysis also employs a three-classification system based on skeletal Class I, II, and III in the horizontal direction, sharing the basic diagnostic framework with Sassoni’s classification. In the vertical direction, however, it classifies faces into three types: Brachy facial, Meso facial, and Dolicho facial, which roughly correspond to Sassoni’s short frame, medium frame, and long frame types.

Therefore, the Sassouni classification is an effective framework for understanding maxillofacial morphology as a comprehensive analysis method that can integrate horizontal and vertical elements while maintaining consistency with other diagnostic systems such as Ricketts analysis. However, clear criteria and standardization for its application have not been established, and the thresholds and weightings for cephalometric analysis items have not been unified, so interpretations may differ among evaluators.

Furthermore, the Sassouni classification is based on adult populations and does not fully reflect the morphological changes specific to the growth period. When applying it to children, adjustments and standardization that take growth characteristics into account are required.

To address this issue, developing an AI classification model for pediatric craniofacial morphology and enhancing the objectivity and reproducibility of diagnosis is expected to enable adaptation to morphological changes associated with growth, contributing to early treatment intervention and optimization of treatment plans.

The objective of this study is to develop an AI model for classifying maxillofacial morphology in children using lateral cephalometric analysis and to evaluate its classification performance.

A part of this study was previously presented as a poster at the 84th Annual Meeting of the Japanese Orthodontic Society in 2025 [9].

## 2. Materials and Methods

### 2.1. Data Collection

This cross-sectional study included 300 children aged 6 to 10 years (134 boys: 8 years 6 months ± 12 months; 166 girls: 8 years 6 months ± 10 months) who were examined at the Orthodontic Department of Iwate Medical University Uchimaru Medical Center between January 2011 and July 2023. All subjects had complete initial examination records. All participants were in Hellmann’s developmental stage (tooth age) IIIA to IIIB. Patients who had previously undergone orthodontic treatment or who had conditions that could affect maxillofacial morphology during growth, such as cleft lip and palate, or chromosomal abnormalities, were excluded.

This study was approved by the Ethics Committee of Iwate Medical University (Approval Number: 01373).

### 2.2. Training Data Preparation

**Figure 1:**
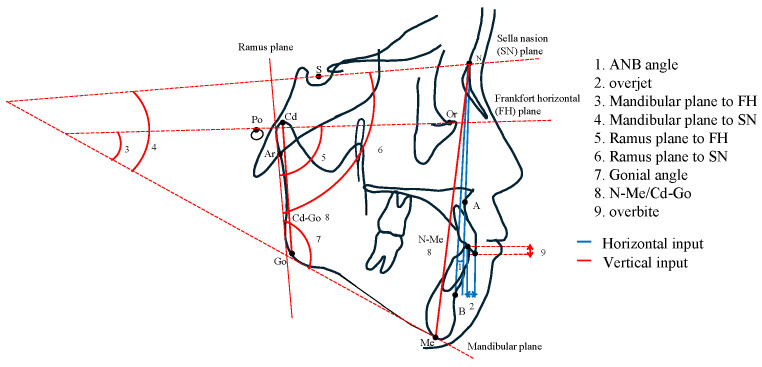
Diagram of cephalometric analysis items used for classification. The blue lines (1, 2) indicate the items used for horizontal classification, whereas the red lines (3–9) indicate the items used for vertical classification.

Lateral cephalograms obtained at the initial examination were analyzed using WinCeph software (Version 11; Rise Corporation, Sendai, Japan). From the 127 analysis items generated, two horizontal parameters (ANB angle and overjet) and seven vertical parameters (mandibular plane to FH, mandibular plane to SN, ramus plane to FH, ramus plane to SN, gonial angle, overbite, and N-Me/Cd-Go) [10,11] were selected as input features for maxillofacial morphology classification (Figure 1). The ANB angle, selected as a horizontal parameter, is the most widely used indicator and is employed in the general Class I, Class II, and Class III classifications.

In vertical classification, the Mandibular plane to FH, Mandibular Plane to SN, Ramus Plane to FH, and Ramus Plane to SN accurately represent the position of the mandible. Together with the Gonial Angle, these are crucial for evaluating mandibular morphology and rotation direction.

Furthermore, N-Me/Cd-Go represents the ratio of anterior facial height to posterior facial height, serving as an auxiliary parameter for vertical classification.

Alongside these items, overjet and overbite, reflecting the occlusal state, were entered as input features.

Calibration was performed using lateral cephalograms with a metal ruler inserted. Scale correction was applied based on the known distance between two points on the ruler, and calibration was implemented in WinCeph. This converted all linear measurements on the cephalometric images to actual physical distances.

Three orthodontists (two instructing physicians and one certified physician, all members of the Japanese Orthodontic Society) classified each patient’s maxillofacial morphology horizontally into skeletal Class I, Class II, or Class III and vertically into short frame, medium frame, or long frame. Combining the three horizontal and three vertical classifications yielded nine morphological patterns (skeletal Class I short frame, skeletal Class II short frame, skeletal Class III short frame, skeletal Class I medium frame, skeletal Class II medium frame, skeletal Class III medium frame, skeletal Class I long frame, skeletal Class II long frame, and skeletal Class III long frame).

The orthodontists classified each patient independently. If two orthodontists agreed on one category and the third differed, a majority decision was applied. If all three selected different categories, the case was discussed until a consensus was reached. These consensus-based classifications were then used as the gold standard classification. Model training and evaluation were performed using 10-fold cross-validation. The 300 patients were randomly divided into 10 subsets. At each iteration, nine subsets were used for model training, and one subset was used for evaluation. The average performance across all iterations was calculated as the final evaluation metric.

### 2.3. AI Model Development

For the Sassouni classification, the Python scikit-learn (sklearn) package (version 1.6.1) was used. Data preprocessing and numerical computations were performed using pandas (version 2.2.2) and numpy (version 2.0.2).

Three machine learning (ML) models were trained and evaluated:

(1) Random forest classifier (RF);
(2) Logistic regression (LR);
(3) Support vector classification (SVC).

**Figure 2:**
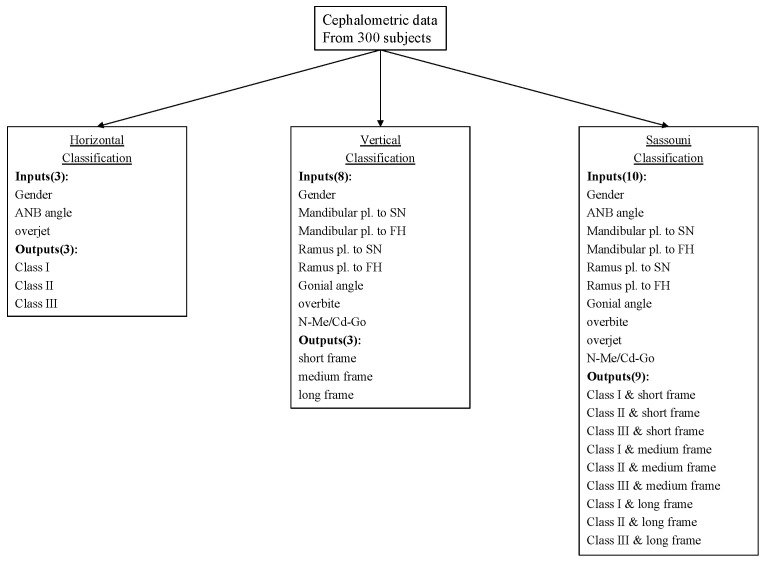
Input and output items of the AI model for horizontal, vertical, and Sassouni classifications. Horizontal classification generated three results from three input items, vertical classification generated three results from eight input items, and Sassouni classification generated nine results from ten input items.

Each model was trained and evaluated using 10-fold cross-validation. Results from each iteration were averaged to assess overall performance. Model classification outputs were then compared with the orthodontists’ classifications (Figure 2).

### 2.4. Analysis of Input Features Importance

The feature importance function in the scikit-learn package was applied to identify which input features most strongly influenced the output of the Sassouni classification model.

## 3. Results

### 3.1. Orthodontist Classification Results

The classification agreement between Observer 1 and Observer 2 was 0.589 according to Cohen’s κ, between Observer 2 and Observer 3 it was 0.603, and between Observer 1 and Observer 3 it was 0.871.

Fleiss’s κ, indicating overall agreement, was 0.684. All these values indicated agreement within an acceptable range.

**Figure 3:**
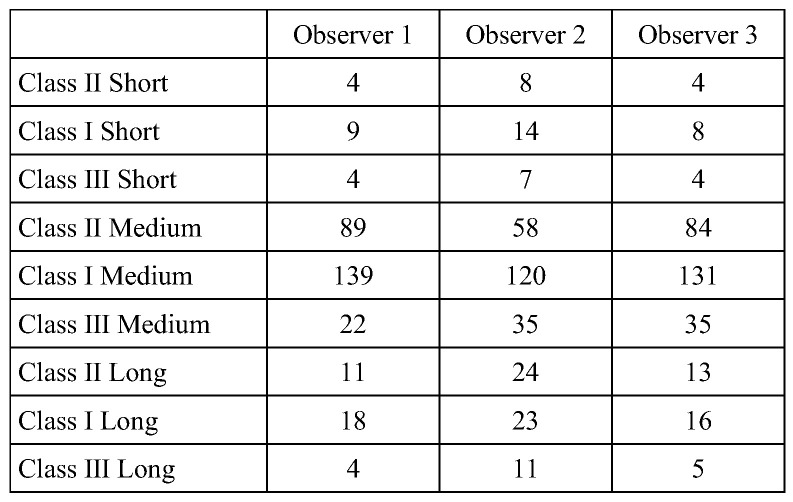
Distribution of Sassouni Classification by Three Orthodontists. The vertical axis represents the nine jaw classifications based on the Sassouni classification, while the horizontal axis indicates each observer (1–3).

First, we present the individual classification results by three orthodontists (Observers 1–3, Figure 3).

**Figure 4:**
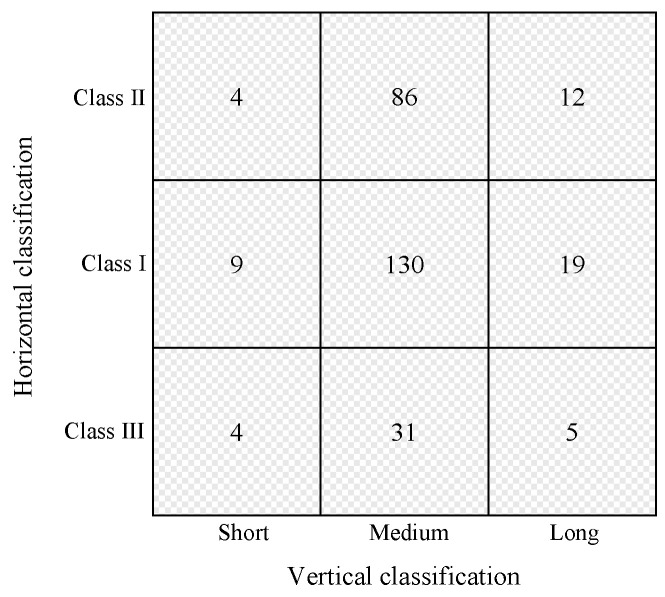
Distribution of orthodontists’ classification results. The vertical axis indicates the horizontal classification, and the horizontal axis indicates the vertical classification.

The distribution of case numbers in the final integrated classification is as follows. Final training data: 4 skeletal Class II short frames, 9 skeletal Class I short frames, 4 skeletal Class III short frames, 86 skeletal Class II medium frames, 130 skeletal Class I medium frames, 31 skeletal Class III medium frames, 12 skeletal Class II long frames, 19 skeletal Class I long frames, 5 skeletal Class III long frames (Figure 4).

### 3.2. Comparison with ML Model Classification Performance

Three ML models (RF, LR, and SVC) from the Python scikit-learn package were tested. Their performance was compared, and the optimal model was identified. The RF model achieved a precision of 0.907 ± 0.051, an F1 score of 0.740 ± 0.160, a sensitivity of 0.750 ± 0.147, and a positive predictive value of 0.741 ± 0.170. Compared with the LR and SVC models, RF demonstrated the highest performance across all evaluation metrics (precision, F1 score, sensitivity, and positive predictive value) (Table 1).

### 3.3. Classification Performance for Horizontal Classification Alone

**Figure 5:**
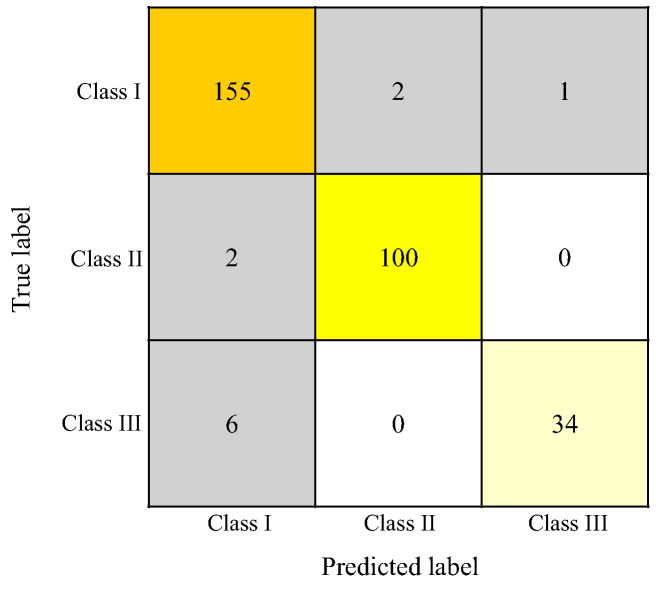
RF confusion matrix for horizontal classifications using k-fold (*n* = 10) cross-validation. The vertical axis represents the orthodontists’ classification results, and the horizontal axis represents the AI’s classification results. Orange to lemon-yellow cells indicate agreement between the orthodontists and AI classifications, whereas gray cells indicate disagreement.

For horizontal classification (Skeletal Classes I, II, and III), the RF model achieved a macro-average accuracy of 0.963 ± 0.031, an F1 score of 0.948 ± 0.044, a sensitivity of 0.937 ± 0.053, and a positive predictive value of 0.971 ± 0.030 (Table 2). The concordance rate was highest for skeletal Class II (0.98), followed by skeletal Class III (0.97), and lowest for skeletal Class I (0.95) (Table 3). Misclassification occurred in 11 patients, most commonly involving skeletal Class III cases being classified as skeletal Class I (Figure 5).

**Figure 6:**
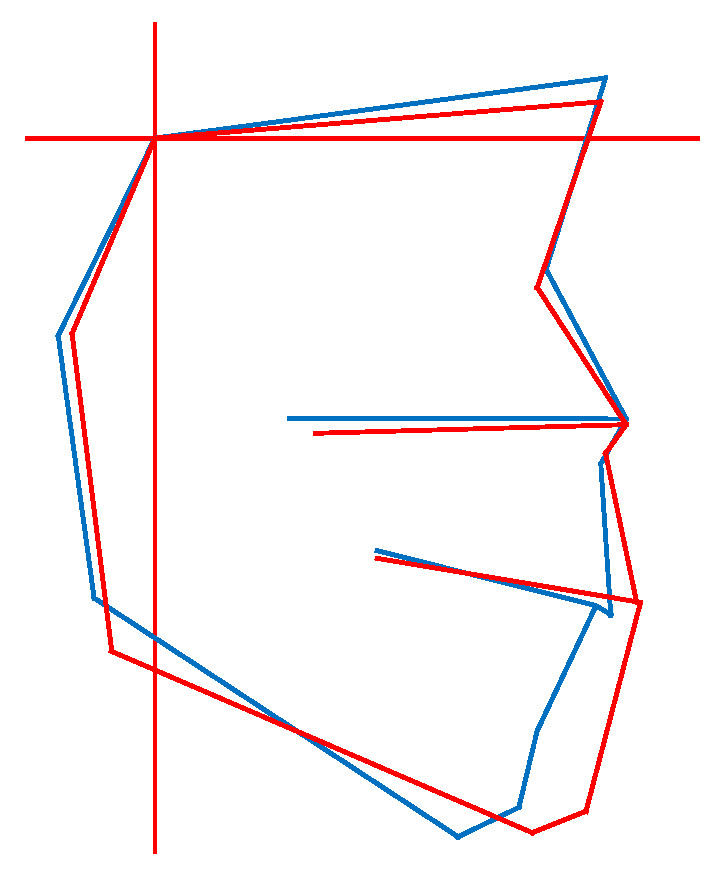
Example of misclassification in horizontal classification. The red line shows the patient’s profilogram, while the blue line shows the average profilogram of individuals with normal occlusion at the same stage of growth and development. In this case, the ANB angle is located near the classification boundary (between Class I and Class III).

In horizontal classification, misclassifications tended to occur frequently when the ANB angle was close to the classification boundary (e.g., near the threshold between Class I and Class III, Figure 6).

### 3.4. Classification Performance for Vertical Classification Alone

**Figure 7:**
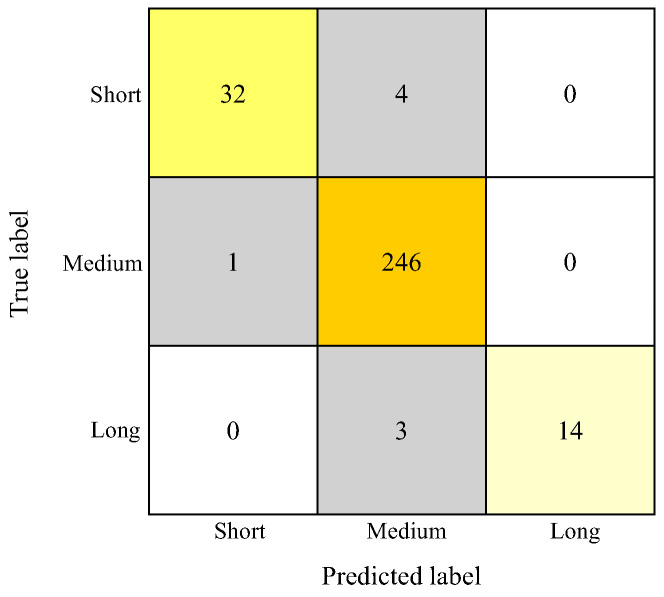
RF confusion matrix for vertical classifications using k-fold (*n* = 10) cross-validation. The vertical axis represents the orthodontists’ classification results, and the horizontal axis represents the AI’s classification results. Orange to lemon-yellow cells indicate agreement between the orthodontists and AI classifications, whereas gray cells indicate disagreement.

For vertical classification (Short frame, Medium frame, Long frame), the RF model achieved a macro-average accuracy of 0.973 ± 0.025, an F1 score of 0.937 ± 0.055, a sensitivity of 0.915 ± 0.075, and a positive predictive value of 0.983 ± 0.028 (Table 2). The concordance rate was highest for short frame (1.00), followed by medium frame (0.97) and long frame (0.97) (Table 4). Misclassification occurred in 8 patients, with the most frequent error being short frame cases misclassified as medium frame, followed by short frame cases misclassified as long frame (Figure 7).

**Figure 8:**
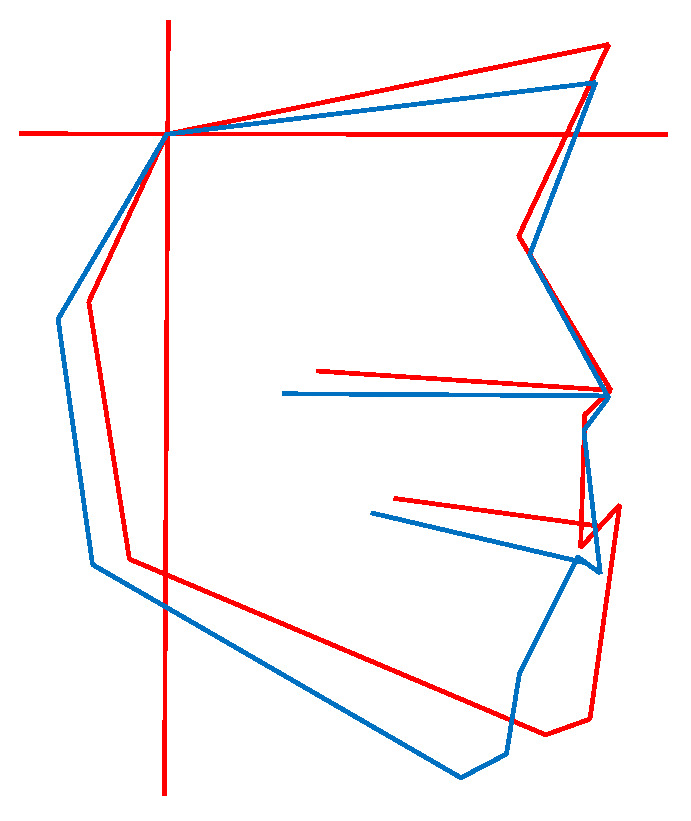
Example of misclassification in vertical classification. The red line shows the patient’s profilogram, while the blue line shows the average profilogram of individuals with normal occlusion at the same stage of growth and development. In this case, the patient’s profile is displaced relative to the reference plane.

In vertical classification, misclassification was more likely to occur in cases where there was a misalignment between the reference plane and the patient’s plane (Figure 8).

### 3.5. Classification Performance for Sassouni Classifications

**Figure 9:**
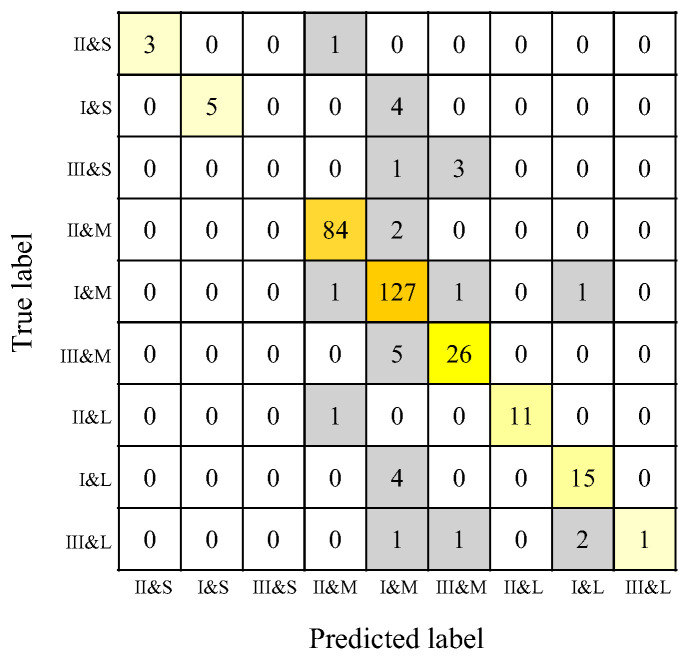
Sassouni classification confusion matrices for the RF model using 10-fold cross-validation. The vertical axis represents the orthodontists’ classification results, and the horizontal axis represents the AI’s classification results. I, II, and III denote skeletal Class I, skeletal Class II, and skeletal Class III, respectively; S, M, and L denote short frame, medium frame, and long frame, respectively. Orange to lemon-yellow cells indicate agreement between the orthodontists and AI classifications, whereas gray cells indicate disagreement.

Concordance rate, reproducibility, and F1 score were calculated for each of the nine Sassouni classifications using the RF model. The highest concordance rates were observed in skeletal Class II short frame, skeletal Class I short frame, skeletal Class II long frame, and skeletal Class III long frame. The lowest concordance rate was observed for the skeletal Class III short frame. The classifications with the highest reproducibility were skeletal Class II medium frame and skeletal Class I medium frame, while skeletal Class III short frame had the lowest reproducibility. The highest F1 score was observed in skeletal Class II medium frame, and the lowest in skeletal Class III short frame (Table 5). A total of 28 patients were misclassified. Of these, 2 patients were misclassified in both the horizontal and vertical components, 11 were misclassified only in the horizontal component, and 15 were misclassified only in the vertical component. Common errors included skeletal Class I short frame misclassified as skeletal Class I medium frame, skeletal Class III short frame misclassified as skeletal Class III medium frame, skeletal Class III medium frame misclassified as skeletal Class I medium frame, and skeletal Class I long frame misclassified as skeletal Class I medium frame (Figure 9).

### 3.6. Feature Importance

**Figure 10:**
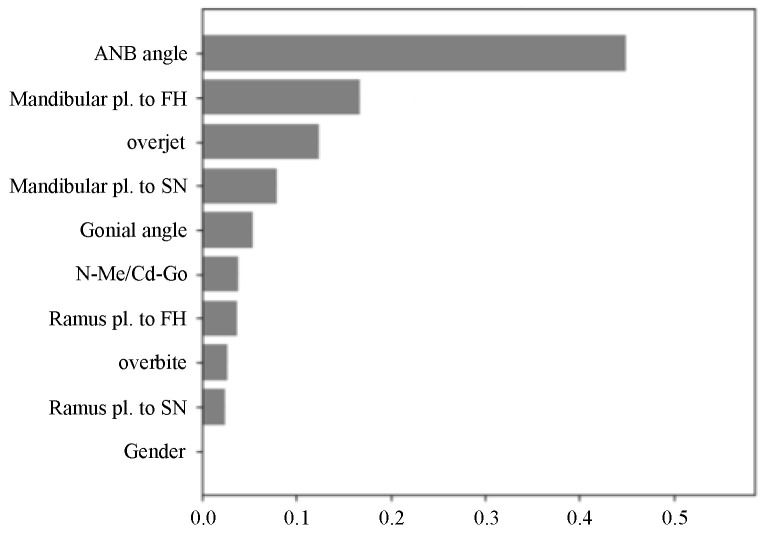
Feature importance of gender and nine cephalometric variables in Sassouni classification. The vertical axis lists the features, and the horizontal axis indicates their relative importance.

Feature importance analysis was performed to identify the influence of input variables on the Sassouni classification model (Figure 10). The ANB angle had the greatest impact, followed by the mandibular plane to FH and overjet. In contrast, the patient’s sex had the least impact on model predictions.

## 4. Discussion

### 4.1. AI Model Machine Learning Algorithm

In this study, we developed an AI model to classify the maxillofacial morphology of children in the growth and development phase and compared the performance of three ML algorithms: RF, LR, and SVC. Data from 300 children were used to train the AI model, and classification performance was evaluated. Results showed that the RF model outperformed the other models across all metrics—precision, F1 score, sensitivity, and positive predictive value (PPV). The RF model constructs multiple classification rules (decision trees) based on randomly selected features and integrates them for prediction. This method can handle non-linear data and complex patterns and is relatively tolerant to overfitting. In our study, the analysis items followed either normal or lognormal distributions, with some showing regression relationships with each other. If classification can be achieved using only a few analysis items, a linear model may suffice. However, because classification in this study required combinations of multiple items, the RF model, which is well-suited for complex data, demonstrated superior performance [12,13,14,15,16].

The LR model showed the second-best performance. It calculates probabilities from input features and applies them to binary classifications. LR is computationally efficient, accurate, and easy to interpret. However, it assumes linear relationships among input variables. In this study, the response variable was maxillofacial morphology, and cephalometric items were explanatory variables, which differed from standard binary classification settings. This may have contributed to lower performance than RF. Nevertheless, increasing the number of samples in smaller groups (e.g., skeletal Class III short frame, skeletal Class III long frame, skeletal Class II short frame) could potentially improve LR performance despite its binary-classification design. The small sample size in certain groups likely reduced their classification performance [17,18,19,20,21].

SVC performance was the weakest. SVC maps data into a higher-dimensional space and classifies it by identifying a separating hyperplane with the maximum margin. Although kernel functions can enhance performance with non-linear data, model accuracy is highly dependent on appropriate kernel and parameter selection, which can be difficult. Inappropriate settings may significantly reduce classification performance. Moreover, SVC relies heavily on support vectors near decision boundaries; thus, performance declines when there is high variability or class imbalance. Because our dataset included uneven group sizes, this likely contributed to the weaker classification results [22,23,24].

Although RF demonstrated high classification performance, this study only performed k-fold cross-validation as internal validation, and its reproducibility on external data remains unverified. Therefore, the possibility of overfitting cannot be completely ruled out, and future work requires confirmation of generalization performance through external validation using independent datasets.

On the other hand, while LR and SVM excel in computational efficiency and parameter interpretability, they have limitations in classifying pediatric maxillofacial morphology, which involves non-linear changes during growth and interactions among multiple factors. In contrast, the decision tree-based RF is advantageous as it can flexibly capture complex non-linear relationships while suppressing overfitting. It is considered a method that combines high performance and practicality for real-world clinical use.

Therefore, the results of this study suggest that RF may be the most practical and reproducible algorithm for automated jaw classification, and its further application and development are anticipated.

### 4.2. RF Model Horizontal and Vertical Classification

For horizontal classification, 11 cases were misclassified, indicating strong overall performance. However, skeletal Class III cases were frequently misclassified as skeletal Class I. This may be explained by the role of the ANB angle in horizontal classification. Borderline values of the ANB angle (around 2 ± 2°) are interpreted differently among researchers [25,26,27], and borderline values may have led to misclassification in this study. Additionally, the small number of skeletal Class III cases in the training dataset may have reduced the model’s ability to accurately recognize this group. Since participants were in Hellmann’s stages IIIA to IIIB (pre-adolescence), skeletal features, particularly mandibular protrusion, were not yet fully pronounced. Because mandibular growth accelerates during adolescence, ANB angle differences become more apparent later, but at this developmental stage, the feature distribution was relatively narrow. This developmental factor may have contributed to misclassifications. For vertical classification, 8 cases were misclassified, with most errors involving short frames or long frames being classified as medium frames. Overall, vertical classification performance was superior to horizontal classification. This may be due to vertical classification relying on seven analysis items, compared with only two for horizontal classification. However, data imbalance was also present: 247 cases were classified as medium frame, while only 36 were short frame cases, and 17 were long frame cases. Such an imbalance may have influenced erroneous classifications.

In horizontal classification, misclassification tended to occur more frequently in cases where the ANB angle was located near the classification boundary (e.g., near the threshold between Class I and Class III). In these cases, the ANB angle is prone to slight variation due to the position of the S-point or N-point, or the rotation direction of the mandible, and may not always accurately reflect the actual skeletal relationship (Figure 6).

In contrast, in vertical classification, errors in setting the reference plane (FH plane or SN plane) and individual variations caused angle measurements like MP-FH and MP-SN to shift vertically. This resulted in classifications crossing the boundary line. It was confirmed that even slight differences in the tilt of the reference plane could be a factor in misclassification (Figure 8).

### 4.3. Sassouni Classification

Using the Sassouni classification, 28 cases were misclassified (11 horizontal, 15 vertical, and 2 in both). Overall classification performance decreased compared with horizontal- or vertical-only models. Common misclassifications included skeletal Class III as Class I and short frame as medium frame. Ueda et al. [28] applied AI classification to adult patients and reported clearer morphological distinctions, with an even distribution of 220 patients across groups. Their RF model achieved horizontal precision of 0.986 ± 0.011, vertical precision of 0.855 ± 0.037, and overall Sassouni precision of 0.823 ± 0.060. In contrast, our study targeting children achieved slightly lower horizontal precision (0.963 ± 0.031) but higher vertical and overall precision compared with the adult study. This may be attributable to our larger sample size (300 children), high-quality training data, and the large number of skeletal Class I medium frame cases (130 patients), which likely enhanced learning and improved classification accuracy.

### 4.4. Input Feature Importance

Feature importance analysis indicated that ANB angle, mandibular plane to FH, and overjet were the most influential features, reflecting their relevance to anteroposterior jaw relationships and mandibular rotation. In contrast, sex had the least impact, likely because boys and girls in the pre-adolescent phase show minimal differences in maxillofacial size. Additionally, distance measurements reflecting size were not included, and N-Me/Cd-Go was expressed as a proportion. Similarly to prior adult studies, the ANB angle was the most important feature, followed by the mandibular plane to FH. However, slight differences were observed in other feature rankings between children and adults, reflecting morphological variation by age group. Similarly to adults, the high feature importance of ANB angle and FMA is likely because the training data encapsulates the diagnostic reasoning patterns of clinicians. In actual diagnosis, a stepwise process is common: first, the ANB angle is used to assess the anterior–posterior relationship, followed by the FMA to evaluate the vertical tendency. Subsequently, consistency with supplementary measurements is verified, and if inconsistencies are found, the diagnosis is reconsidered. This conditional and hierarchical clinical decision-making structure was learned by the model, resulting in the high contribution of the primary indicators, ANB angle and FMA.

Furthermore, because most patients in our dataset were classified as skeletal Class I medium frame, the AI model may not have had sufficient training data for borderline cases. Expanding underrepresented categories through targeted data collection may help improve classification performance.

Future efforts should focus on balancing group sizes and incorporating additional feature quantities to enhance model accuracy and generalizability.

## 5. Conclusions

The RF-based AI model demonstrated high accuracy in classifying pediatric maxillofacial morphology, outperforming both LR and SVC. These results indicate that AI-based Sassouni classification can be performed with high precision in children, potentially providing objective and reproducible diagnoses for maxillofacial morphology during growth stages. AI-based automatic classification is considered useful for reducing inter-clinician variability in assessments and for early identification of growth direction and skeletal tendencies. Therefore, this approach supports the auxiliary use of AI in orthodontic diagnosis and has the potential to contribute to diagnostic standardization and improved early treatment planning.

Future studies should further verify the generalizability and clinical applicability of the proposed model through external validation using multi-center datasets.

## Figures and Tables

**Table 1:** Comparison of the performance of Sassouni classification using random forest classifier (RF), logistic regression (LR), and support vector classification (SVC) with k-fold (*n* = 10) cross-validation.

	RF	LR	SVC
Accuracy	0.907 ± 0.051	0.837 ± 0.057	0.770 ± 0.055
F1 score	0.740 ± 0.160	0.623 ± 0.132	0.507 ± 0.102
Sensitivity	0.750 ± 0.147	0.631 ± 0.116	0.534 ± 0.102
Positive predictive value (PPV)	0.741 ± 0.170	0.641 ± 0.155	0.499 ± 0.113

**Table 2:** Comparison of the performance of RF models for horizontal and vertical classifications.

	Horizontal Classification	Vertical Classification
Accuracy	0.963 ± 0.031	0.973 ± 0.025
F1 score	0.948 ± 0.044	0.937 ± 0.055
Sensitivity	0.937 ± 0.053	0.915 ± 0.075
Positive predictive value (PPV)	0.971 ± 0.030	0.983 ± 0.028

**Table 3:** Performance of horizontal classification outputs in the RF model.

Classification	Precision	Recall	F1 Score	Support
Class I	0.95	0.98	0.97	158
Class II	0.98	0.98	0.98	102
Class III	0.97	0.85	0.91	40
Accuracy			0.96	300
Macro avg.	0.97	0.94	0.95	300
Weighted avg.	0.96	0.96	0.96	300

**Table 4:** Performance of vertical classification outputs in the RF model.

Classification	Precision	Recall	F1 Score	Support
Short	1.00	0.82	0.90	17
Medium	0.97	1.00	0.98	247
Long	0.97	0.89	0.93	36
Accuracy			0.97	300
Macro avg.	0.98	0.90	0.94	300
Weighted avg.	0.97	0.97	0.97	300

**Table 5:** Performance of Sassouni classification outputs in the RF model.

Classification	Precision	Recall	F1 Score	Support
Class II Short	1.00	0.75	0.86	4
Class I Short	1.00	0.56	0.71	9
Class III Short	0.00	0.00	0.00	4
Class II Medium	0.97	0.98	0.97	86
Class I Medium	0.88	0.98	0.93	130
Class III Medium	0.84	0.84	0.84	31
Class II Long	1.00	0.92	0.96	12
Class I Long	0.83	0.79	0.81	19
Class III Long	1.00	0.20	0.33	5
Accuracy			0.91	300
Macro avg.	0.84	0.67	0.71	300
Weighted avg.	0.90	0.91	0.89	300

## Data Availability

The data presented in this study are available on request from the corresponding author. The data are not publicly available due to privacy.

## Abbreviations

The following abbreviations are used in this manuscript:

RF	Random Forest
LR	Logistic Regression
SVC	Support Vector Classification
